# Experiences of hospital allied health professionals in collaborative student research projects: a qualitative study

**DOI:** 10.1186/s12913-022-08119-7

**Published:** 2022-06-01

**Authors:** Rebecca L. Angus, H. Laetitia Hattingh, Kelly A. Weir

**Affiliations:** 1 Allied Health and Rehabilitation Services, Gold Coast Hospital and Health Service, 1 Hospital Boulevard, Southport, Queensland 4215 Australia; 2 School of Health Sciences and Social Work, Griffith University, Gold Coast, Queensland Australia; 3 Medical Services, Clinical Governance and Research, Gold Coast Hospital and Health Service, 1 Hospital Boulevard, Southport, Queensland 4215 Australia; 4 School of Pharmacy and Medical Science, Griffith University, Gold Coast, Queensland Australia

**Keywords:** Research capacity building, Allied health, Healthcare performance, Research supervision

## Abstract

**Background:**

Active engagement in research by healthcare organisations and clinicians is associated with improvements in healthcare performance. Barriers to research engagement by clinician allied health (AH) professionals include competing priorities from high clinical workloads, lack of research skills and confidence, and lack of supportive research relationships. Collaboration with universities on joint clinical research projects is well recognised as a means of building health service research capacity. Research projects undertaken by students as part of their qualifying degree represent one such opportunity. However, there are few reports evaluating these collaborations from the health service perspective.

**Methods:**

A qualitative study using semi-structured interviews and thematic analysis to explore the experiences of AH professionals in the co-supervision of students completing research placements as part of their professional degree course.

**Results:**

Fourteen health service employees from six allied health disciplines described collaborations on research projects with 24 students from four different universities. Student placements and projects varied widely in length of placement, extent of collaboration, supervision structure and study design. Three overarching themes were identified in the AH professional co-supervision experience: 1) Professional growth; 2) Mismatch with expectations; and 3) Focus on the student. Project outcomes were categorised from the health system perspective. These were 1) Healthcare performance improvements, including local increases in staff clinical practice knowledge and wider contributions to the evidence base; 2) Research capacity gains within the health service, including research knowledge and skill development, collaborative linkages and opportunity for future research; and 3) Staff-centred outcomes including increased job satisfaction.

**Conclusions:**

This study demonstrates the potential for AH professional supervision of students on research placements to contribute to healthcare performance improvements and research capacity gains within health services, alongside providing personal benefits for the AH professionals involved. Early consultation with a health service-employed research specialist may support health professional and student learning, team collaboration and project coordination for these student projects.

**Supplementary Information:**

The online version contains supplementary material available at 10.1186/s12913-022-08119-7.

## Background

Clinical and health-services research is fundamental to providing an evidence-base for practice and effective high-quality healthcare. The process of attaining new knowledge to improve clinical practice extends from the laboratory to clinical settings and the subsequent translation into practice. The involvement of medical, nursing and allied health (AH) professionals who possess well-developed research skills is essential at various levels of this process [1–3]. Indeed, basic research skills including the ability to ask clinical questions and acquire, critically appraise and interpret research evidence are core competencies in evidence-based practice for all health professionals [4]. Within the university setting, research education may be research-led, where academics utilise their research expertise to inform their teaching, or research-based, where students develop skills through direct involvement in research activities. Integration of these two strategies may aid development of higher-level skills and learners who are more independent, autonomous and critical [5]. Thus, in addition to coursework in research methods, supervised research projects are offered as electives or incorporated into the allied health degree programs of many universities. These projects differ from those undertaken by postgraduate research-only students (e.g. Masters by Research or Philosophy, or Doctoral programs) and are typically of shorter duration (weeks to months) and with less student ownership of the project in terms of development of research questions and/or study design. While often university-based, these projects may also be conducted in collaboration with community partners including health services. An evaluation of the dietetics programs of two London universities found projects undertaken in collaboration with health services provided a more positive research experience for students. Both students and academic (university) supervisors consistently rated these projects as being more likely to change practice, lead to further research and be disseminated at professional conferences and published in peer reviewed journals than studies conducted purely within university faculties [6].

Active engagement in research by healthcare organisations and clinicians is associated with improved healthcare performance [7, 8]. However, numerous barriers to clinician involvement in research have been identified. For AH professionals these include funding pressures, time and competing priorities from high clinical workloads, lack of research skills and confidence, unhelpful workplace culture and systems for research, and lack of supportive research relationships [9–11]. Strengthening research capacity by addressing these barriers is an identified priority in enabling AH professionals to contribute to improving healthcare systems performance. Various frameworks to guide research capacity building amongst AH professionals have been developed to this effect [12, 13]. Collaboration with universities via involvement in joint research projects, or through supervision and mentoring of clinicians undertaking research higher degrees is well recognised as a means of establishing health service research capacity [14]. Research projects undertaken by students as part of their qualifying degree have also been identified as a potential research capacity building opportunity for Australian hospital services [15]. Collaboration on such projects may create opportunities for clinicians to build research skills, develop linkages and partnerships and ensure that research is close to practice – three principles of building research capacity in health services [16]. However, there is limited literature evaluating such projects from the health service perspective. Survey responses from health service staff involved in supervision of dietetic student research projects indicated that the opportunity to expand own research area, share expertise and access data or facilities were the main benefits gained from their involvement [6]. A study of physiotherapy student research projects undertaken at the University of Toronto examined project impacts based on the perceptions of the lead student supervisor. These included both academics and clinicians, with stated main impacts on clinical practice and own research capacity, followed by knowledge translation (e.g., publication, presentation, further studies), education and health policy. Between 45 and 75% of advisors agreed the projects helped them build research skills [17].

As a publicly funded teaching hospital and health service in Australia, our organisation is actively engaged in student instruction and learning. Within the allied health disciplines, in addition to providing clinical placements this extends to providing opportunities for students undertaking research projects as part of their professional degree. However, the contribution made by such projects to health service improvement and their potential for building health service research capacity has not been well examined. The objective of this study was to explore the expectations and experiences of AH professional staff within a large tertiary health service in the supervision of university students on collaborative research placements and to identify any beneficial outcomes of these from the health service perspective.

## Methods

This exploratory, qualitative study used semi-structured interviews to explore the experiences of health service employed AH professionals in the supervision of students on clinical research placements. Research projects were limited to those undertaken as part of students’ professional degree qualification programs (Bachelor or Master professional qualification, excluding students undertaking a higher degree by research). All staff with an allied health qualification (audiology, dietetics, occupational therapy, pharmacy, physiotherapy, podiatry, psychology, social work or speech pathology) who had supervised a research student in their professional field within the past five years were eligible for participation. Ethical approval was granted by the Gold Coast Hospital and Health Service Human Research Ethics Committee and performed in accordance with the Declaration of Helsinki. The consolidated criteria for reporting qualitative research were followed in the reporting of this study [18].

### Setting

Gold Coast Hospital and Health Service (GCHHS) is a tertiary health service in southeast Queensland comprised of two hospitals (~ 1200 beds), a day hospital and community outpatient hubs providing publicly funded inpatient and outpatient health services to a local population of 650,000 people. Within GCHHS Allied Health, a small team of research fellows are employed to support research capacity building across the service. Provision of clinical practice supervision for students on placement is written into State-wide health practitioner role descriptions for all allied health disciplines [19]. Student clinical placements are managed at the Health Service departmental discipline level on a contract basis with partner universities. In contrast, involvement with student research placements is mostly undertaken by staff on an ad hoc basis as opportunity and interest arises.

### Positionality of researchers

The study team comprised career researchers with professional qualifications and registration in allied health (RA-dietetics, LH-pharmacy, KW-speech pathology) and research doctorates. All were female, employed within GCHHS as Allied Health Research Fellows and held conjoint (KW) or adjunct (RA, LH) appointments with a partner University. An insider perspective was taken, based on previous involvement supporting clinicians in the supervision of student research projects in formal and/or informal capacities. The team members had not previously worked together on any project and all had training and experience in qualitative interviewing and analysis methodology. Participants were aware of the research teams’ professional backgrounds, and in some cases, the interviewer was previously known to the participant. Where a student collaborative project had been supported by a research fellow, participant interviews were conducted by a researcher external to that project. Participants were informed their responses were confidential, would not affect their employment and that all information would be de-identified for publication.

### Data collection

Recruitment was via direct email approach from the researchers, with potential participants purposively identified through discussion with allied health discipline leads, research staff and via snowball sampling. A study participant information sheet was provided and those who agreed to be interviewed signed a consent form. An interview guide was developed based on a review of the literature, the professional experiences of the research team and using the consolidated framework for implementation research (CFIR) [20] as a guide (supplementary 1). Specific questioning around reasons for student project involvement, project outcomes and future intentions was included. Demographic and student project details were collected via survey prior to interview. For each participant, a single, semi-structured interview was conducted face-to-face in a private room with only interviewer and participant present. Field notes were completed immediately after interviews to capture non-verbal content and allow contextualisation of data, along with a researcher journal to enhance reflexivity. Interviews were audio recorded, transcribed verbatim and checked against recordings for accuracy, but not returned to participants for inspection. Recruitment continued until no further consenting participants could be identified.

### Data analysis

Thematic analysis followed the six-steps of data familiarisation; initial code generation; identification of potential themes; review of themes; defining/naming themes; and writing up findings [21]. Six interview transcripts were inductively coded by two researchers each, with discussion between all three team members to form an initial thematic framework. A single researcher (RA) then coded all transcripts, with coded excerpts assessed against team determined themes (LH or KW) and any discrepancies resolved by discussion. Project outcomes relayed in interviews were grouped and categorised according to value from the Health Service perspective, using previous literature as a guide. NVivo (QSR International Pty Ltd) was used to facilitate data organisation and coding.

## Results

### Study participants

In total, 16 AH professionals were identified as recently having supervised students on research placements and were invited for interview. Two additional AH professionals were identified who were on extended parental leave. Consent to participate was received from 14 with no response from the remainder. Interviews lasted 21–58 minutes and were conducted between March and July 2021. The sample included 11 clinicians and three allied health research fellows from six AH professional disciplines (Table 1). Audiology, psychology and speech pathology departments indicated that their clinical staff had no involvement in supervising student research projects within the study scope. The gender ratio of participants reflected that of AH professionals within the health service while the higher than workforce-average age and employment level was likely explained by inclusion of research involvement within role descriptions for staff employed at senior levels [22]. Clinician participants had collaborated with 24 students from four different universities who worked on 18 distinct projects or project phases across a range of study designs (Table 1). Research fellows discussed their experiences in both formal co-supervision of student projects, and in providing informal assistance to clinicians collaborating on student projects, both during and after student placement periods.

**Table 1:** Participant demographics and student project details

Participant demographics	n	Student and project details	n
**Total participants**	14	**Total students**^c^	24
Female	12	**Universities**	4
Male	2	**Student disciplines**	
**Level of experience**^a^		Dietetics	10
Base grade HP	1	Occupational Therapy	6
Senior HP	5	Pharmacy	1
Advanced/Team Leader	5	Physiotherapy	6
**Allied Health discipline**		Social Work	1
Dietetics	5		
Occupational Therapy	2	**Discrete projects/phases**^c^	18
Pharmacy	1	Retrospective clinical audit	3
Physiotherapy	2	Survey	2
Social Work	1	Qualitative study	3
Research Fellows^b^	3	Prospective observational	3
**Age, years**	41.5 (29–55)	Systematic/literature review	4
**Years since graduation**^a^	14.9 (7–22)	Randomised control trial^d^	2
**Years clinical experience**^a^	14.0 (7–20)	Laboratory study	1

Data given as counts, or average years (range) where indicated. ^a^Clinician participants only, excludes research fellows. ^b^Professional disciplines of allied health research fellows were speech pathology, dietetics and pharmacy. ^c^Students/projects supervised by clinician participants. Research fellows interviewed were involved in some of the projects listed, as well as various other projects supervised by clinicians outside of the study participants. ^d^Students contributed to protocol and/or resource development to support planned future randomised control trial. *HP* Health Practitioner.

### Description of student projects and roles

There was substantial variation in student projects, both between and within allied health disciplines and universities. Some students worked on standalone projects, while others made contributions to larger projects, such as collecting data for a specific time period in a longer study. Fifteen projects had individual student allocations, and three projects had students working in groups of two, three or four. Student research involvement ranged from taking part solely in data collection, to active participation in numerous other project activities such as protocol development, ethics application, data analysis and dissemination of results. Concordant with this, the timeframe for student involvement ranged widely. Some students were assigned block placements of six weeks to six months duration, others had split placements where involvement with the project was conducted over the course of 12 months. In the latter, project aspects such as protocol and ethics application development were typically worked into university course work, followed by blocks of time for data collection and analysis at later stages. In all cases, taking the work to the publication stage necessitated additional work by university academics, health service clinicians and/or students after the completion of the research placement.

In most cases, the original idea or research question was generated by a health service clinician and was pitched to a university in response to an expression of interest seeking opportunities for student projects. Thereafter, the extent of collaboration between clinician and university academics in project conduct varied widely. In three projects the clinicians had little involvement with the project and/or student after suggesting the original study idea, while for another four projects students worked on studies managed within the health service with little or no university input. The remainder were more collaborative, with genuine involvement in study conduct from both university staff and health service clinicians.

There was also variation in the roles played by health service staff in direct student supervision. Usually, students had academic supervision from university staff to assist with management of their course requirements, although in one case the university provided no academic support, even of a cursory nature. Allied health research fellows embedded within the health service provided project support in either formal or informal roles in all but five of the 18 projects. In some, allied health research fellows were named project members on the student project, while in many other projects clinicians sought informal advice and support during or after the student placements for project management, navigating research relationships or assessing final data quality and planning for ‘what next’.

Generally, participants described their student collaborative projects as successful, although some described negative aspects. When questioned about their motivation for involvement in student projects, AH professionals indicated that they saw this as a means to progress research work more quickly, as an opportunity for learning about research, because they viewed student supervision as part of their role in the health service, or a combination of these reasons.

### Themes of participant experience

Exploration of the experiences of health service staff identified three main themes in student project supervision comprising 1) Professional growth; 2) Mismatch with expectations; and 3) Focus on the student.

1. Professional growth

All clinician participants experienced professional growth as a result of supervising students on research placements. Even clinicians who expressed some dissatisfaction with their experience acknowledged benefits from their involvement. For the majority, gains in research capacity were evident. This included learnings in specific methodological processes for particular study designs (e.g. survey design, conduct of systematic review or qualitative data analysis techniques), as well as more general research process skills including collaborative practice and management of research teams, understanding of the ethical approval processes, and research dissemination skills such as scientific writing and conference presentation.

*All the elements of conducting research that I really had no idea about how to do well … doing ethics or running protocols or just – just all the stuff in behind the scenes about papers and research that you don’t know until you’ve got experience. That was really daunting... But then, as it turned out, the people organising the research set things in place and then got help with it with statisticians and things like that. P10, Dietitian.*

For research fellows, involvement in student projects provided another way to enact their roles in building health service research capacity, supporting clinicians who might otherwise lack time to participate in research, and simultaneously building their own professional skills.

*With every single student I gain more experience as a supervisor and that not only helps me with future students, but it also helps me mentoring clinician researchers as well. P11, Research Fellow.*

Other gains in research capacity included formation or reaffirmation of collaborative linkages supporting the opportunity for future research. Clinician participants also mentioned increased motivation for involvement in research and obtaining results that could form the basis for future studies. Several participants had supervised consecutive students across multiple phases of a study area or mentioned plans to do this. In addition, participants experienced growth outside of research-related facets of their profession. Many increased their clinical practice knowledge as a result of study involvement, and for some, working with students also prompted positive realisation of the extent of their own clinical knowledge and competence. Involvement in student research projects also afforded development of widely transferable skills such as communication, teamwork, project management and conflict resolution, and attainment of personal benefits including job satisfaction, curriculum vitae development and the opportunity to meet role expectations for student supervision and/or research conduct.

2. Mismatch with expectations

For many participants, aspects of their experience of research student supervision did not meet their original expectations, even in cases where they felt the project was overall successful. Several participants had anticipated that students would have higher capabilities than they exhibited. In particular, the extent of clinical skills needed for conducting the specified research tasks. For example, clinicians expected students to have more competency identifying patient eligibility, extracting patient data from clinical records and interpreting this for accuracy and study relevance according to inclusion criteria. In two cases, clinician supervisors expended substantial time after completion of the placement period to clean and/or re-extract data collected by students in order to ensure the study results were valid and useable. In a related aspect, other participants referred to the students they worked with being of especially high competence, recognising that this was an important factor in the success of their projects.

*I think we were expecting too much of the students or I suppose we didn’t know what their level of understanding would be. Because we assumed that having done their clinical placement, they are technically qualified to practise. Yeah, so the areas that we were seeing in their data collection for the clinical skills in the research activity we were sort of floored that it was that poor. P2, Dietitian.*

For some participants, the extent of the additional workload imposed by taking on supervision of a research student was unexpected. The level of clinician involvement and the activities undertaken by the clinician was often determined as the project progressed, with universities attempting to accommodate the desires of their clinician collaborators. Several participants identified this as a positive, indicating they appreciated this flexibility. One participant described negotiations with university academics around the extent of time and involvement given to the student project:

*At the beginning, I’d been trying to be really careful to not agree to too much … they were trying to do whatever we wanted and at the beginning, we were like we can only give this much and they were like you don’t have to give anything, just whatever you want to give and then it evolved. P5, Occupational Therapist.*

Many projects successfully maximised the complementary skills of both research academics and clinicians, alongside student labour contributions, to deliver project outcomes and resultant publications that would not have been possible for either acting alone. However, some clinicians felt that their specialised clinical expertise was not valued by their academic collaborators. They became concerned that interpretations of study results might contribute inaccurate evidence to a field, spur further studies that would constitute research waste or reflect poorly on the health service that they represented.

*It would have been really nice had we been in a more even perhaps playing field of a relationship, that there would have been that mutual respect to say, ‘hey, clinicians have concerns, let’s not do this this way’ because they must know what the system is that we are needing to operate within.*

In a few cases, the study undertaken deviated from the idea or research question originally proposed by the clinician. Occasionally, disagreements about project ownership arose creating problems in planning for continuing studies or in agreements about authorship order. None of these projects had health service research fellow team members involved in the initial project establishment, although these staff later provided formal and/or informal support to clinicians to negotiate these issues. The clinicians involved had not anticipated the power imbalances that arose and reflected that in future they would only participate in such collaborations where a health service employed research fellow was also involved.

*I needed [Research Fellow] in the room in order to get my point across sometimes, you know? So they were using their power differentials a bit too.*

For a few projects, the university academic supervisor appeared to lack the necessary capability to fulfill their expected role in providing research guidance. In these cases, the clinician supervisors had approached research fellows from the health service for assistance in bringing the project back on track or to completion either during the student placement, or afterwards in attempts to salvage data and justify the time, financial costs and effort expended. One research fellow described the experience of joining a research collaboration already in progress, to support research capacity building and role clarification for the health service clinician who provided the idea for a systematic review to support practice:

*There was no formal search done that we could see, there was no use of [systematic review software], there was no joint title and abstract review. So I was very confused as to what they were doing … they weren’t following a best practice of actually doing a systematic review … A lot of the stuff was not up to a standard that we’d be happy to put our name to. P13, Research Fellow*

3. Focus on the student

Many clinician supervisors focused their discourse about project involvement on the student experience. When asked generally about outcomes from the projects, it was only after prompting that several participants appeared to recognise their own research learnings and acknowledge these as project outcomes. Instead, clinicians placed central importance on student-related outcomes, citing student attainment of high thesis/placement marks and/or prestigious graduate employment, along with presentations or first author publications by students. When asked about their contributions to the research project, clinicians often did not mention the impact of their clinical skills and knowledge. Rather, they focussed on their attention to the emotional needs of the student, their efforts to ensure students took clinical learnings from the project to support them in future work as a clinician and discussed the extra work they took on to ensure students met course requirements. Describing the experience with students whose research placement was to develop resources for use in a planned randomised control trial, one clinician noted:

*I really like to give back to students, because it’s not that long since I was there. So, I really think it’s great exposure for students to see how clinics work. Especially the area that I work in because it’s quite a specialised area. P4, Occupational Therapist.*

Another clinician stated:

*I was quite conscious that I actually had to meet her learning goals, as a student, as opposed to what the research goals of delivering a publication in a particular space. So, it wasn’t about, let’s just get to the end goal and that’s what we need to do. We had to tick a lot of boxes to say that you were, could actually walk away and do this again, on your own. P9, Social worker.*

While research fellow participants were more focused on clinician rather than student outcomes, they also reported feeling pride in their students’ achievements and described feeling stress around obtaining timely approvals to enable students to conduct their projects within university prescribed timeframes. In projects where differences of opinions had occurred between academic and clinician supervisors, care had been taken to shield students from these concerns.

### Collaborative student project outcomes

Beneficial outcomes from the health service perspective were grouped into three overarching categories (Table 2). All clinician participants experienced outcomes across at least two of these categories. Research capacity gains within the health service were particularly apparent, and evident for ten of the 11 clinician participants. The two research fellows who had formal roles in co-supervising student projects indicated they saw these collaborative projects as a means to build clinician research capacity in a non-threatening manner. In addition to the development of specific methodological knowledge and wider research skills, the value of linkages formed during projects was evident. Subsequent to their student project involvement, two clinician participants enrolled in PhD courses, taking their previous student project co-supervisor as their own academic supervisor. At time of writing, seven (39%) of the student projects described by clinician participants had resulted in peer reviewed journal articles, with manuscripts in preparation for another four more recently completed projects.

**Table 2:** Outcomes of student research projects contributing to health service excellence

Outcome category	Representative quotes
1) **Healthcare performance improvements**	
**Local**	
Increased clinical practice knowledge of staff	*I learned, in particular about this topic; so because I had to review the student’s work, and I was the principal reviewer of the contents of the survey, so I had to let that – that forced me, or motivated, me to read – to go back to the literature and read and learn and remember some of the things that I thought I knew well, but I had to refresh myself. So it definitely helped my personal knowledge, just the clinical knowledge, and I already made use of some of that knowledge in my day-to-day practice. P1, Pharmacist* *It’s actually given me a lot of knowledge that I wouldn’t have otherwise had, and it does give you a perspective in your clinical practice …*. *It did give a bit of an answer to our question like whether it was feasible or not, or even under what conditions it’s feasible. It gave us like a bit of information about the practical applications of it. P10, Dietitian*
Provision of evidence base to support current processes or practice change improvements	*Wanting to know if patients were having positive outcomes. So you sort of have the feeling that it might be beneficial what we’re doing clinically, but with that, the actual proof, so knowing that we could then get the answers to that, to see what effect and what impact we’re having on patients. …. Just the knowledge of the effectiveness of the different treatments. P7, Dietitian*
**Wider**	
Peer-reviewed publication and/or conference presentations	*We’ve got something more published on the [Intervention] than we had before and it’s on a different angle than other people had looked at, so it’s relevant for a lot of other HHSs [hospital and health services]. P5, Occupational Therapist*
Contribution to guidelines/health policies	*The systematic review … I feel good about that. I suppose the fact that we have contributed to a massive gaping hole that [existed when] we started out, that’s one thing …. It was cited in the [clinical area International] Guidelines. P2, Dietitian*
2) **Research capacity gains within the Health Service**	
Research knowledge and skill development in individual staff	*I knew that I was going to be learning at the same time she [the student] was going to be learning. Yeah, it was very handy, doing it that way. P9, Social Worker* *I have a better understanding on the methodology for research. I have a better understanding of the challenges. I had a lot of help from the others, but I wasn’t aware how time-pressing and complicated the ethics process is and so on. P1, Pharmacist* *There’s been a huge learning curve … even non-research skills like conflict management have been developed as well. I suppose that is probably an aspect of working within a team, in a research team. P2, Dietitian*
Collaborative linkages formed or reaffirmed	*The collaborations sort of create – can develop into further projects down the track. P7, Dietitian*
Opportunity for future research	*It motivated me to look at other projects, and maybe focus a little bit more on research in the future. P1, Pharmacist* *It was a project I was very interested in, and I just don’t want it to stop now that the Honours has been done and dusted. I want to keep on going. P14, Dietitian.*
3) **Staff-centred outcomes**	
Job satisfaction	*I do enjoy the research. I also enjoy being a clinician, so it’s hard to do both. But yeah, usually try and make the most of opportunities that come up so I can do both. P3, Physiotherapist* *I liked the - that sort of building some connections and relationships with the university and the students and I suppose even just from a supervisor point of view is watching the student learn those skills and develop and all of that as well. P12, Dietitian*
Meeting role expectations	*I’m in a senior clinical position and there’s probably some expectation with having a PhD that you continue to do research. So then this was a way that I can – if someone said, ‘what research have you been participating in?’, it was something that I could put forward and say, ‘we’ve been looking at this and doing this’ without spending all my clinical time on it. P8, Physiotherapist*
Curriculum vitae development	*I got a publication, I got a few more different things, I can say I co-supervised an honours student, so I’ve got that now as an experience. P5, Occupational Therapist*

Few project collaborations had formal memoranda of understanding detailing investigator roles, contributions and expectations for the parties involved, although for some projects agreements about authorship order on resulting publications had been put in place prior to students starting. No clinician participants described discussing the personal outcomes they hoped to obtain with their university academic collaborators.

## Discussion

In healthcare organisations, a research culture is associated with improved healthcare performance [4]. This includes improved organisational efficiency, lower patient mortality rates, higher levels of both staff and patient satisfaction, and reduced staff turnover [3]. Our study demonstrates that collaborations between health services and universities for student research placements can provide benefits for health services aligning with some of these outcomes. Such collaborations can provide satisfaction for AH professionals employed in health services through opportunities for professional development, workplace enjoyment and fulfillment, and enabling role expectations to be met in manners suited to individual preferences. They can also contribute to healthcare performance improvements. Evidence suggests that research engagement by clinicians engenders greater research utilisation [8]. At a local level, increased and up-to-date knowledge of the specific research topic, as mentioned by several participants in this study, better enables clinicians to implement optimal care for their patients. This may also translate to greater receptivity to using other new research findings in practice [8]. Several projects examined here also had impact at a broader level, through contributions to the evidence-based literature and citation in international clinical area guidelines. AH professionals in academic roles are more highly incentivised to publish compared to those working clinically, which may have contributed to dissemination success. Publication rate (39%) was similar to that reported for allied health student research projects in other regions in pharmacy (USA; 42.3%) [23] and physiotherapy (Canada; 44.5%) [24]. The health service-university collaboration assists in enabling student research that is close to practice and provides evidence that is relevant to the needs of clinicians, health services and the patients they serve. Moreover, staff authorship can contribute to institutional reputation. Health services held in high regard for their research culture may be linked to the ability to attract and retain high quality clinicians, which may further benefit patient care [8].

Strengthening research capacity in AH professionals is a priority in enabling their contribution to healthcare system improvements [12, 25]. Our study identified research capacity gains as a key outcome for the health service, with collaboration on student research projects enabling AH professionals to overcome some of the identified barriers to clinician involvement in research [9–11]. Most prominently, this included the development and extension of research skills, with clinicians gaining confidence and experience in research methodology, planning, delivery, management, collaboration and/or dissemination. Collaboration with universities has long been encouraged as a means of extending health service research capacity, with AH professionals being encouraged to reach out to academics to progress clinically relevant studies [14, 26, 27]. Student projects may offer clinicians a non-threatening means to establish such linkages and test the waters for future collaborations. Although some progressed no further than the initial student project, several were extended into subsequent projects with new students, and some AH professionals later enrolled in research higher degrees under their former co-supervisor. Finding time amongst busy clinical workloads is an oft reported barrier to AH professional research. However, perceived advantages relating to clinician time from student conduct of data collection and other laborious research tasks were not always realised when balanced against training and supervision requirements.

To the best of our knowledge, this is the first qualitative study providing an in-depth description of the experiences of AH professionals employed within a health service in the supervision of students conducting research projects as part of their qualifying degrees. All participants experienced professional growth in some aspects of clinical, research or other general workplace skills as a result of their involvement. However, although most felt their student projects were successful overall, many indicated their expectations for the collaboration were not matched by the reality of the experience.

Numerous participants commented upon the influence of the capability of individual students on research project progress. Supervision of student clinical placements is part of all AH professional role descriptions in public health services in our region, [19] and AH professionals are generally aware of the limited clinical ability of these students who are still developing their professional practice skills. However, this did not always translate to foreseeing limitations in the accurate collection or interpretation of clinical data by students on research placements. Research project completion is a requirement of allied health degrees offered by some universities, while for others is offered as an elective component, sometimes only available to high performing students. Given the time and work put into these collaborative projects by health service AH professionals and the potential additional cost to later take staff off-line from clinical duties to remedy student errors, the aspect of student capability should be carefully considered in project planning. This should be discussed early on in negotiations with university collaborators.

Our findings, and those of previous studies, [9] indicate that clinicians can struggle to find time to complete research whilst managing busy clinical workloads. The labour provided by students, and often their academic supervisors, meant that many of these projects were able to progress further than would have been possible if conducted solely within the health service. The responsibility felt by clinicians to enable students to complete projects within tight timeframes also contributed. Clinicians were focused on achieving positive outcomes for students, and indicated that to support this, they had worked at levels that would be unsustainable over longer periods. Conducting research to support evidence-based practice is a goal important to both the university sector and public health services [25]. The latter have a key role in providing clinical practice training to students to support future workforce needs. However, in establishing supervisory roles in collaborative student research projects, consideration should be made of the primary goal of each supervisor’s institution - universities in providing student education, and health services in providing patient care.

Participants described some projects in which problems had arisen. These related to design and conduct of the research, conflict around interpretation of results from the academic or clinician viewpoint, ownership of continuing projects and authorship on resulting publications. Typically, this occurred in projects without involvement of a health service research fellow team member, with these called upon later by the health service clinician supervisor to help manage arising issues. Where possible, it may be prudent for clinicians to seek support from a research specialist based within their health service in the early stages of project planning and consider their formal inclusion on the project team. These staff have the knowledge of research practice and standards required to ensure appropriate research conduct within clinical settings [28] and can advocate for clinicians if the need arises. Further, they have knowledge of the requirements for research within the ethical and governance systems of health services, which may differ from those which academics working within university systems are familiar with.

It is evident that AH professional clinician co-supervision of student research projects can bring benefits for health services as well as for universities and students. However, the AH professional participants in this study tended to focus their attention on student outcomes with few projects having defined potential benefits that might be realised by the health service at the outset. It was notable that although many clinicians cited a desire to learn about research as a motivation for co-supervising a research student, none described documenting learning goals for themselves, or included these in project planning with academic collaborators. A more detailed analysis of the barriers and facilitators to building research capacity in health services via these student research projects may be warranted. As a starting point to optimising outcomes for health services, clinicians may seek to define clear personal learning goals along with other potential benefits for the health service using Table 2 as a guide. These should be discussed with academic collaborators during project planning stages. Early consultation with a health service employed research fellow may assist clinicians in compiling realistic research and professional development goals. Research fellows may also be able to assist in project planning by moderating expectations around student capability and advise clinicians on negotiating project ownership, role clarification and expectations, authorship considerations and division of supervisory responsibilities with academic collaborators. In some cases, their specialist research expertise may also be necessary for the design and/or conduct of high-quality studies appropriate to the clinical setting, or in relation to health service ethical requirements.

### Strengths and limitations

A strength of the study was representation by participants from various allied health professions, who described their experiences in collaborating with academic staff and students from multiple universities across projects of a range of study designs, making the results more broadly applicable outside of our specific setting. The sample size was limited by the number and availability of eligible participants, but never-the-less exceeded the 12 interviews that has been shown to be sufficient for theoretical data saturation in qualitative studies [29]. The insider perspective provided an intimate understanding of contextual factors and influences, supporting effective probing during interviews to elicit richer information from participants, and with organisational and professional knowledge reducing the likelihood of participant responses being misunderstood. However, the personal experiences of team members in supervising research student projects may have affected interpretation of the experiences described by participants. We endeavoured to manage the intrinsic bias arising from this through vigorous discussion, sharing the observations, interpretations and understanding of each team member. Maintenance of a research journal provided a reflective space recording change and development of ideas over the course of interviews and data analysis, and an audit trail of the process.

## Conclusions

This study has demonstrated the potential for AH professional supervision of students on research placements to contribute to healthcare performance improvements and research capacity gains within the health services, alongside personal benefits for the AH professionals involved. Further work is needed to identify the facilitators and barriers to achieving optimal outcomes and supporting clinician research capacity development from these collaborations. However, early consultation with, or inclusion of a health service-employed research fellow on supervisory teams may assist in the smooth running of these student projects and help maximise potential benefits for the health service.

## Supplementary Information

**Additional file 1.**

## Data Availability

The datasets generated during this study are not available due to the sensitive and personal nature of the information contained. Data may be available upon justified request from the corresponding author with restrictions and following ethical approval.

## Abbreviation

AH professionals: Allied health professionals

## References

[1] Butler D (2008). Translational research: crossing the valley of death. Nature..

[2] Department of Health and Social Care. Executive Office (Northern Ireland), The Scottish Government and Welsh Government. Saving and improving lives: The future of UK clinical research delivery [Internet]. 2021. [cited 2022 May16]. Available from https://www.gov.uk/government/publications/the-future-of-uk-clinical-research-delivery.

[3] Harding K, Lynch L, Porter J, Taylor NF (2017). Organisational benefits of a strong research culture in a health service: a systematic review. Aust Health Rev.

[4] Albarqouni L, Hoffmann T, Straus S, Olsen NR, Young T, Ilic D (2018). Core competencies in evidence-based practice for Health professionals: consensus statement based on a systematic review and Delphi survey. JAMA Netw Open.

[5] Huet I (2018). Research-based education as a model to change the teaching and learning environment in STEM disciplines. Eur J Eng Educ.

[6] Whelan K, Thomas JE, Madden AM (2007). Student research projects: the experiences of student dietitians, university faculty members, and collaborators. J Am Diet Assoc.

[7] Boaz A, Hanney S, Jones T, Soper B (2015). Does the engagement of clinicians and organisations in research improve healthcare performance: a three-stage review. BMJ Open.

[8] Hanney S, Boaz A, Jones T, Soper B (2013). Health services and delivery research. Engagement in research: an innovative three-stage review of the benefits for health-care performance.

[9] Borkowski D, McKinstry C, Cotchett M, Williams C, Haines T (2016). Research culture in allied health: a systematic review. Aust J Prim Health.

[10] Harvey D, Plummer D, Nielsen I, Adams R, Pain T (2016). Becoming a clinician researcher in allied health. Aust Health Rev.

[11] Wenke R, Noble C, Weir KA, Mickan S (2020). What influences allied health clinician participation in research in the public hospital setting: a qualitative theory-informed approach. BMJ Open.

[12] Harris J, Grafton K, Cooke J (2020). Developing a consolidated research framework for clinical allied health professionals practising in the UK. BMC Health Serv Res.

[13] Matus J, Walker A, Mickan S (2018). Research capacity building frameworks for allied health professionals - a systematic review. BMC Health Serv Res.

[14] Ward EC, Elphinston RA, Wall LR, Schwarz M, Gordon GE (2018). Research engagement and activity in an allied Health workforce insights into departmental and Organisational strategies. J Allied Health.

[15] Alison JA, Zafiropoulos B, Heard R (2017). Key factors influencing allied health research capacity in a large Australian metropolitan health district. J Multidiscip Healthcare.

[16] Twelvetree T, Suckley J, Booth N, Thomas D, Stanford P (2019). Developing sustainable nursing and allied health professional research capacity. Nurse Res.

[17] Lacey C, Scodras S, Ardron J, Sellan R, Garbaczewska M, O'Brien KK (2018). Retrospective review of student research projects in a Canadian master of science in physical therapy Programme and the perceived impact on Advisors' research capacity, education, clinical practice, knowledge translation, and Health policy. Physiother Can.

[18] Tong A, Sainsbury P, Craig J (2007). Consolidated criteria for reporting qualitative research (COREQ): a 32-item checklist for interviews and focus groups. Int J Qual Health Care.

[19] Queensland Industrial Relations Commission. Health Practitioners and Dental Officers (Queensland Health) Award – State 2015, 2018 State Wage Case Reprint. State of Queensland; 2018.

[20] Damschroder LJ, Aron DC, Keith RE, Kirsh SR, Alexander JA, Lowery JC (2009). Fostering implementation of health services research findings into practice: a consolidated framework for advancing implementation science. Implement Sci.

[21] Braun V, Clarke V (2006). Using thematic analysis in psychology. Qual Res Psychol.

[22] Queensland Health. Gold Coast Health workforce strategy 2019–2024 [Internet]. Southport: State of Queensland; 2019. [Cited 2022 February 15]. Available from: https://www.goldcoast.health.qld.gov.au/.

[23] Assemi M, Ibarra F, Mallios R, Corelli RL (2015). Scholarly contributions of required senior research projects in a doctor of pharmacy curriculum. Am J Pharm Educ.

[24] McEachern BM, Winningham I, Wood K, Tang J, VanDerWeide T, O'Brien KK (2020). Factors associated with publication of research projects from a Canadian master of science degree Programme in physical therapy. Physiother Can.

[25] Queensland Health. Optimising the allied health workforce for best care and best value: A 10-year Strategy 2019–2029 [Internet]. Southport: State of Queensland; 2019. [Cited 2022 February 02]. Available from: https://www.health.qld.gov.au/ahwac/

[26] McLaughlin MM, Skoglund E, Bergman S, Scheetz MH (2015). Development of a pharmacy student research program at a large academic medical center. Am J Health Syst Pharm.

[27] Pighills AC, Plummer D, Harvey D, Pain T (2013). Positioning occupational therapy as a discipline on the research continuum: results of a cross-sectional survey of research experience. Aust Occup Ther J.

[28] Wenke RJ, Ward EC, Hickman I, Hulcombe J, Phillips R, Mickan S (2017). Allied health research positions: a qualitative evaluation of their impact. Health Res Policy Syst.

[29] Guest G, Bunce A, Johnson L (2006). How many interviews are enough?: an experiment with data saturation and variability. Field Methods.

