# Supplementary material for: Experiences of hospital allied health professionals in collaborative student research projects: a qualitative study
Source: BMC Health Serv Res. 2022 Jun 1;22:729. doi: 10.1186/s12913-022-08119-7 (PMC9161454; doi:10.1186/s12913-022-08119-7)
Supplement: Supplementary file 1 — Additional file 1. [file 12913_2022_8119_MOESM1_ESM.docx]

**Semi-structured interview guide**

Please tell me about your involvement in student research supervision.

Provide a brief description of the last student project that you were involved in supervising.

How did the opportunity to be involved in student supervision come about?

*Prompts:*

- *Whose idea was the project?*
- *Where did the idea to have a student conduct the project come from?*
- *How did you feel about having a student conduct the project?*
- *Was there any other option for getting this project done? If so, why did you choose the student option?*
- *Who or what else influenced your decision to be involved in a student research project?*

What did you hope would be gained from involvement in this student project supervision?

*Prompts:*

- *Personally: research skills, partnerships, resources etc*
- *For department/health service/clinical area*
- *For University/student*

What do you feel was gained from your involvement in research student supervision?

*Prompts:*

- *Personally: research skills, partnerships, resources etc*
- *For department/ health service/clinical area*
- *For University/student*
- *How would the project have differed if you had not been involved? (what was your contribution?)*

Were there negatives to your involvement in the supervision of the student research placement?

*Prompts:*

- *Impact on clinical work, other duties, own time etc*

What challenges did you experience throughout the project and how did you address them?

*Prompts:*

- *Processes, roles, research conduct, relationships*

Did you have a clear expectation of what your involvement would be when you started the student supervision collaboration?

*Prompts:*

- *In supervision? In direct research?*
- *Did it differ from what your involvement eventually was? How?*
- *Was there any written agreement in place?*

Please expand on the support you received in supervising the student project?

*Prompts:*

- *What support? (time in lieu, resources/software, supervision instruction, research building skills - in which areas? project design, ethics, data collection, analysis, dissemination)*
- *Who from? (collaborators/Uni, research office, department, other staff)*

Can you tell me about communications between different members of the research team?

*Prompts:*

- *With Uni, student, others. How? (face-to-face, email, phone).*
- *Who/how was this organized? Was it sufficient?*
- *How outcomes communicated to stakeholders?*

Do you consider this project as successful? Why?/Why not?

*Prompts:*

- *Consider changes in practice, service improvement, support further studies, contribute to evidence (publication, conference), student degree result.*
- *What might have helped give a better outcome?*

At what point in the research cycle did the student finish?

*Prompts:*

- *Did their involvement extend beyond their formal completion date?*
- *What other resources/support/funding/input was needed to get the project to completion/publication?*

Did you enjoy the experience?

*Prompts:*

- *What aspects did you enjoy?*
- *What aspects did you not enjoy?*
- *Which aspects were easy?*
- *What aspects were challenging?*

Would you recommend other clinicians be involved in research student supervision?

*Prompts:*

- *What recommendations do you have for them?*
- *Would you supervise another student?*

------------------

For staff with extensive experience with collaborative supervision of research students:

Ask about the most recent student projects, and collect information for each area as per interview guide below as it arises, with additional questions/prompts such as

- Does that differ from the way you proceeded in previous projects?
- Was your experience different in the earlier student projects you were involved with?
